# Perspective: Cell therapy, SARS‐CoV‐2, COVID‐19, and James Lind

**DOI:** 10.1002/acg2.88

**Published:** 2020-05-21

**Authors:** Robert P. Gale

**Affiliations:** ^1^ Centre for Haematology Research Department of Immunology and Inflammation Imperial College London London UK

The severe acute respiratory syndrome‐coronavirus‐2 (SARS‐CoV‐2) pandemic and resultant coronavirus infectious disease‐2019 (COVID‐19) create challenges and opportunities for physicians specializing in cell therapy and hematopoietic cell transplants. Recipients of these therapies are at high‐infection risk because immune suppression and bone marrow failure from these therapies and their consequences such as cytokine release syndrome (CRS) and chronic graft‐*versus*‐host disease (G*v*HD) and from their underlying disease.

Little is known about the impact of the SARS‐CoV‐2 pandemic on cell therapies such as chimeric antigen receptor (CAR)‐T‐cell therapy. Because CAR‐T‐cell therapy uses autologous T cells there is no risk of SARS‐CoV‐2‐infection from a donor. However, the recipient may be infected before, during, or after the therapy and unlikely to fare well if given immune suppressive drugs such as cyclophosphamide and fludarabine preinfusion. There may also transportation restrictions if the processing center is remote from the recipient, risk of infection from healthcare provider, family members, etc. Also, because CAR‐T‐cell recipients are at high risk of CRS, a complication shared with severe COVID‐19 and the combination could be fatal. Although there are reasonably convincing data of safety and efficacy on interleukin‐6 antagonists in CRS related to CAR‐T‐cell therapy, most data suggest that this approach is ineffective in COVID‐19‐related pneumonia.^1^, ^2^ The US Food and Drug Administration (FDA) has approved a phase‐3 placebo‐controlled trial of tocilizumab in COVID‐19 pneumonia (https://clinicaltrials.gov/ct2/show/NCT04320615); I await results. Elsewhere, my colleagues and I review the possible role of mesenchymal stromal cells in COVID‐19‐related acute respiratory distress syndrome.^3^ Others are studying placenta or umbilical cord‐derived natural killer (NK)‐cells (https://www.europeanpharmaceuticalreview.com/news/116794/us‐researchers‐to‐study‐stem‐cell‐therapy‐in‐covid‐19‐patients/). Preliminary results of phase‐2 trails are being reported, but data are mostly uninterpretable regarding efficacy with appropriate controls. If cell‐therapy‐based approaches prove safe and effective, operationalizing this will involve hematologists, especially those in cell therapy and/or hematopoietic cell transplantation.

There are few data about SARS‐CoV‐2‐infection and COVID‐19 in persons with hematological cancers. My Chinese colleagues and I studied several aspects of this in Wuhan, epi‐center of the SARS‐CoV‐2 pandemic and in Hubei province. In one study in > 500 persons with chronic myeloid leukemia (CML), we found only five cases of COVID‐19.^4^ Although this case rate was substantially higher than the case rate in normals in Hubei province, it is obviously not a serious issue in persons with CML. Risk factors included exposure to someone infected with SARS‐CoV‐2, no complete hematological remission (CHR), initial presentation as advanced leukemia despite responding to therapy and, possibly, receiving a third‐generation tyrosine kinase inhibitor (TKI).

In another study we compared > 100 hospitalized persons with hematological disorders in two hospitals in Wuhan to similar numbers of healthcare providers in the same hospitals. We found similar COVID‐19 case rates, about 10%, but a much higher case fatality rate in the hospitalized hematology patients, about > 60%. We were unable to identify prognostic or predictive covariates in the hematology patients for developing or dying from COVID‐19 suggesting the need for careful infection surveillance and possible protective isolation.^5^

In the third study, we sought to determine safety and efficacy of corticosteroids in persons with COVID‐19 and other severe coronavirus infections including SARS‐CoV and MERS‐CoV in a meta‐analysis of 11 studies in 5247 subjects.^6^ Corticosteroid use was associated with delayed virus clearing with no significant reduction in the risk of death. Hospitalization duration was prolonged and the use of mechanical ventilation increased. These data caution against using corticosteroids in persons with severe COVID‐19. The three studies I cite are published or in press in *LEUKEMIA* and available with open access at the NATURE *LEUKEMIA* website (https://www.nature.com/leu/).

We also used data from our experience at a transplant center in Hangzhou to suggest guidelines for transplant activities during the SARS‐CoV‐2 pandemic.^7^ These guidelines are mostly similar to those proposed by others such as the European Bone Marrow Transplant Group (EBMT; https://www.ebmt.org/ebmt/news/coronavirus‐disease‐covid‐19‐ebmt‐recommendations‐update‐march‐23‐2020) and American Society for Transplantation and Cellular Therapy (ASTCT; https://www.journalofclinicalpathways.com/news/astct‐releases‐interim‐guideline‐transplantation‐during‐covid‐19), but are based on our *real‐time* experience. None of these so‐called guidelines are evidence‐based and should be used with this caveat in mind. Guidelines for infection control are available from the European Hematology Association (EHA) and also available at https://www.nature.com/leu/.

Finally, the rush to try new therapies in persons with SARS‐CoV‐2‐infection and COVID‐19 under the banner *there is nothing to lose* is, I believe, a mistake. Trying new therapies without the evidence of safety and efficacy can cost rather than save lives. With the SARS‐CoV‐2 pandemic and COVID‐19 we have a sudden disruption of 267 years of progress in the evolution of evidence‐based medicine. In Royal Navy, surgeon James Lind may have reasoned *there is nothing to lose* giving two sailors with scurvy two lemons and an orange, but he also gave others cider (not so bad), diluted sulfuric acid (not so good), vinegar, sea water, or a spicy paste as a purgative. Although we lack evidence he had Ethics Committee approval, the pair receiving the lemons and orange improved. We are not told what happened to the others. His discovery resulted in England's mastery over the seas proving it is sometimes better to be lucky than smart. When Captain Cook went on his 1st voyage on the Endeavour to Tahiti in 1768 he carried whiskey mash (not bad), sauerkraut, and a syrup of oranges and lemons.

However, two lemons and an orange are a far cry from suggesting people take chloroquine, hydroxychloroquine, azithromycin, household disinfectant (I prefer 409), and internal UV‐light to prevent or treat SARS‐Cov‐2‐infection or COVID‐19. Based on the advertisements in airplane shopping magazines read awaiting takeoff and unable to use your laptop (remember those days) an interval UV light is more likely to grow hair in your oropharynx than cure SARS‐CoV‐2‐infection. We should not let evidence‐based medicine be hijacked by politicians, charlatans, and the like. Fortunately, most hematologists are sensible and disciplined; certainly readers of *Advances in Cell and Gene Therapy* are. Hopefully, we will get through this pandemic with our scientific principles intact.

## CONFLICT OF INTEREST

None.

## ETHICS STATEMENT

I confirm adhering to the ethical policies of the journal noted on the journal author guidelines page. No ethical approval was required for this Perspective article with no original research data. However, I advise against drinking diluted sulfuric acid.

## References

[1] Kennedy LB , Salama AKS . A review of cancer immunotherapy toxicity. CA Cancer J Clin. 2020;70:86‐104. 31944278 10.3322/caac.21596

[2] Luo P , Liu Y , Qui L , Liu X , Liu D , Li J . Tocilizumab treatment in COVID‐19: a single center experience. J Med Virol. 2020. 10.1002/jmv.25801 PMC7262125 32253759

[3] Hamden H , Hashmi SK , Lazarus HM , Gale RP , Qu W , Fakih RE . Possible role of mesenchymal stromal cells in coronavirus infectious disease‐19‐related severe acute respiratory syndrome. Bone Marrow Transplant. 2020. (in press). 10.1016/j.blre.2020.100742 PMC7425550 32854985

[4] Li W , Wang D , Guo J , et al. COVID‐19 in persons with chronic myeloid leukaemia. Leukemia. 2020 (in press). 10.1038/s41375-020-0853-6 PMC7233329 32424293

[5] He W , Chen L , Chen LI , et al. COVID‐19 in persons with haematological cancers. Leukemia. 2020 (in press). 10.1038/s41375-020-0836-7 PMC7180672 32332856

[6] Li H , Chen C , Hu F , et al. Evidence against use of corticosteroids SARS‐CoV‐2, SARS‐CoV and MERS‐CoV infections. Leukemia. 2020 (in press). 10.1038/s41375-020-0848-3 PMC7199650 32372026

[7] Xiao H , Luo Y , Shi J , et al. Recommendations for hematopoietic stem cell transplantation during the COVID‐19 outbreak: experience and perspective from China. Bone Marrow Transplant. 2020 (in press).

